# No relevant association of kinematic gait parameters with Health-related Quality of Life in Parkinson’s disease

**DOI:** 10.1371/journal.pone.0176816

**Published:** 2017-05-22

**Authors:** Kristina Bettecken, Felix Bernhard, Jennifer Sartor, Markus A. Hobert, Marc Hofmann, Till Gladow, Janet M. T. van Uem, Inga Liepelt-Scarfone, Walter Maetzler

**Affiliations:** 1 Center for Neurology and Hertie Institute for Clinical Brain Research, Department of Neurodegeneration, University of Tuebingen, Tuebingen, Germany; 2 DZNE, German Center for Neurodegenerative Diseases, Tuebingen, Germany; 3 Hasomed, Magdeburg, Germany; 4 Institute for Physiotherapy, Department of Clinical Research, Jena University Hospital, Jena, Germany; Instituto Cajal-CSIC, SPAIN

## Abstract

**Background:**

Health-related Quality of Life (HrQoL) is probably the most important outcome parameter for the evaluation and management of chronic diseases. As this parameter is subjective and prone to bias, there is an urgent need to identify objective surrogate markers. Gait velocity has been shown to be associated with HrQoL in numerous chronic diseases, such as Parkinson’s disease (PD). With the development and wide availability of simple-to-use wearable sensors and sophisticated gait algorithms, kinematic gait parameters may soon be implemented in clinical routine management. However, the association of such kinematic gait parameters with HrQoL in PD has not been assessed to date.

**Methods:**

Kinematic gait parameters from a 20-meter walk from 43 PD patients were extracted using a validated wearable sensor system. They were compared with the Visual Analogue Scale of the Euro-Qol-5D (EQ-5D VAS) by performing a multiple regression analysis, with the International Classification of Functioning, Disability and Health (ICF) model as a framework.

**Results:**

Use of assistive gait equipment, but no kinematic gait parameter, was significantly associated with HrQoL.

**Conclusion:**

The widely accepted concept of a positive association between gait velocity and HrQoL may, at least in PD, be driven by relatively independent parameters, such as assistive gait equipment.

## Introduction

Health-related Quality of Life (HrQoL) is considered the most important factor describing treatment efficacy in patients with chronic (progressive) diseases, such as Parkinson's disease (PD) [1,2]. Not surprisingly, HrQoL measures are used to evaluate disease progression and treatment outcomes [3–5]. One downside of these measures is that they build on a subjective “experience” with a potentially high risk of bias. This is relevant for clinical trials and for the efficient management of chronic diseases, and objective surrogate markers that reflect aspects of HrQoL are urgently needed.

HrQoL models including multiple domains and interactions between these domains represent realistic relationships between the parameters of interest [6,7]. An example of a model that can be used to describe health status (including HrQoL) is the International Classification of Functioning, Disability and Health (ICF) framework, developed by the World Health Organization (WHO) in 2001 [8]. The ICF model includes five interacting domains: body function, activity, societal participation, environmental factors and personal factors. It enables the inclusion of quantitative parameters, such as kinematic gait parameters. This is relevant as wearable sensor techniques are increasingly recognized as a valuable option to evaluate and manage features of chronic diseases. The measurement of these features goes beyond clinical judgment and evaluation, which is often subjective [9,10]. Algorithms can “translate” sensor data into a plethora of clinically relevant parameters, such as rhythmicity, symmetry and regularity [11]. A coherence between sensor-measured Timed-Up-and-Go parameters and HrQoL has already been published [12].

PD can be seen as a model chronic disease for the evaluation of parameters that affect HrQoL. A wide variety of motor and non-motor features can be observed in PD, including gait, mood and cognitive deficits [13]. All these features can affect HrQoL, and there is evidence that axial features (such as gait disturbances [14–17]) in particular lead to decreased HrQoL. Comparable with other chronic diseases, such as multiple sclerosis [18], chronic stroke [19] and osteoarthritis [20], gait difficulties occur even at early stages of PD and progress over the course of the disease [21,22].

Therefore, we investigated the relationship between predefined gait parameters and a widely used HrQoL measure using of the ICF model.

## Methods

### Design and participants

Forty-three PD patients (14 females) from the EGGS study (*Erfassung von Gang- und Gleichgewichtsstörungen*) were prospectively assessed at the ward of the Neurology Department of the University Hospital Tuebingen between 12/2014 and 04/2015. Inclusion criteria were: i) age between 40 and 89 years, ii) diagnosis of PD according to the UK Brain Bank Society criteria [23], and iii) ability to stand stable for at least 30 seconds and walk on even ground for at least 100 meters. The use of a walking aid was allowed. Exclusion criteria were: i) more than one fall per week during the last four months (due to high risk of falling during the assessment) and ii) a Mini Mental State Examination (MMSE) [24] score below 10 points (due to the risk of misinterpretation of the given instructions). The local ethical board of the Medical faculty of the University of Tuebingen approved the study (No. 356/2014BO2). The study was conducted in accordance with the principles of the latest version of the Declaration of Helsinki. All participants signed the informed consent prior to assessment.

### Clinical assessment

All participants underwent a detailed clinical assessment during their medication ON phase, including a medical history and neurological evaluation.

HrQoL was assessed using the Visual Analogue Scale of the Euro-Qol-5D (EQ-5D VAS) [25], an instrument that is widely used to assess HrQoL in the (typically chronically ill) respondent’s immediate situation. The EQ-5D VAS is a validated HrQoL assessment tool in PD [25,26]. A mark must be positioned on a line between 0 (worst imaginable HrQoL) and 100 (best imaginable HrQoL).

In addition, the following tests were performed: the motor part of the MDS version of the Unified Parkinson’s Disease Rating Scale (MDS-UPDRS III) [27], the MMSE [24], a self-developed physical activity questionnaire (adapted from a recent questionnaire that allows the calculation of metabolic equivalents [28]), the Tilburg Frailty Indicator (TFI) [29] (which provides information about the respondent’s living environment, mobility difficulties, weight course during recent months, fatigue and forgetfulness), the Beck’s Depression Inventory II (BDI) [30] and the Lachs-Questionnaire [31] (which assesses functional disability in older patients).

### Gait assessment and data extraction from the sensors

For the gait assessment, participants performed a straight walk of 20 meters on their usual self-selected gait velocity in an at least 3-m-wide obstacle-free corridor. The participants started from a position marked with a line and walked over a similar line at the end of the 20m distance. The exact instruction was as follows: “Please walk with your usual gait speed to a similar line on the floor at the end of the hallway, as you see here in front of your feet. Please stop after you have crossed that line.” Before the assessment, participants were equipped with three statically calibrated sensors (RehaGait®, Hasomed GmbH, Magdeburg, Germany [32]), one located at the lower back and one on each ankle. Each RehaGait sensor has a magnitude of 60x35x15 mm, a weight of 34 g, and includes three axis accelerometers (range ± 16 g), three gyroscopes (range ± 2000°/s), and a magnetometer (range ± 1,3 G). The data was streamed in real-time at 100 Hz to a tablet via Bluetooth. Processing of raw data was performed using validated algorithms [33]. The entire walking distance except the starting step was analysed.

### Statistical analysis

Demographic, clinical, MDS-UPDRS III and sub scores [34–37], kinematic gait and HrQoL parameters are presented as the mean and standard deviation or, in the case of non-normally distributed data, as the median and range or percentage of the total. The following kinematic gait parameters were selected, as they represent relatively independent gait domains [11]: gait velocity, step duration, stride time variability (variability of stride duration) and step asymmetry (asymmetry of swing times between the legs [38]).

Spearman rank correlations were performed, in order to select the most relevant parameters for the block-wise multivariate regression model. This method was also used to evaluate correlation coefficients (r) between all parameters. An r>0.4 between two parameters [39] led to the exclusion of one of the two parameters. Detailed results are shown in S1 Table.

**Fig 1:**
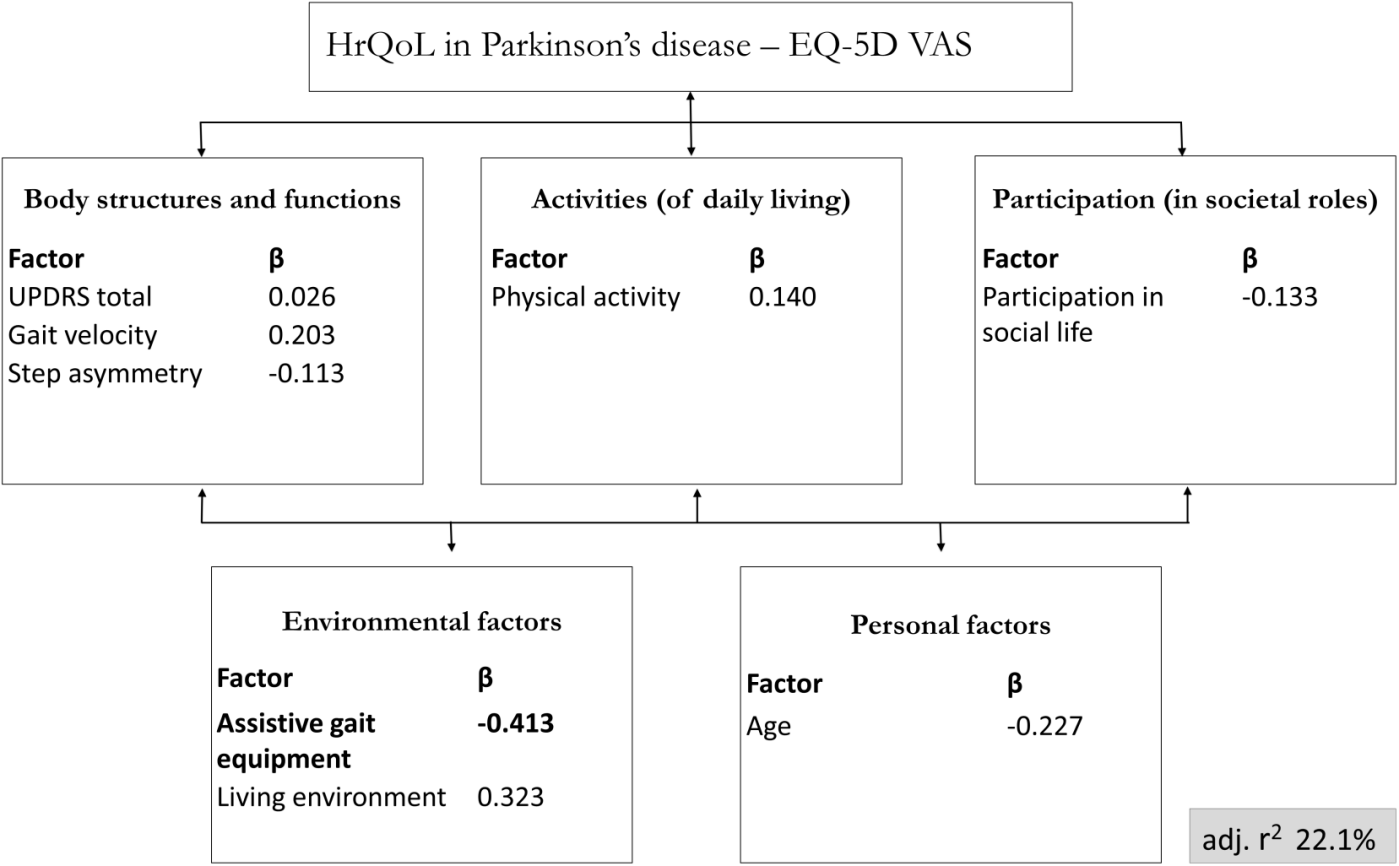
Block wise multivariate regression analysis for HrQoL using the ICF model as framework. Block wise multivariate regression analysis for Health-related Quality of Life (HrQoL) assessment in Parkinson’s disease with consideration of International Classification of Functioning, disability and health (ICF)-relevant parameters, including kinematic gait parameters. Significant β-values are presented in bold. EQ-5D VAS, Visual Analogue Scale of the Euro-QoL-5D; adj. r^2^, adjusted regression coefficient for the entire model.

For the block-wise multivariate regression analysis, kinematic gait parameters were assigned to the body function domain, physical activity (calculated from the metabolic equivalents of task) was assigned to the activities domain, the items “loss of fun” and “loss of interest” of the BDI [30] were assigned to the participation domain, assistive gait equipment (no equipment vs. walking cane vs. wheeled walker) and living alone versus living with others (from the TFI [29]) were assigned to the environmental factors domain, and age was assigned to the personal factors domain of the ICF model. The EQ-5D VAS score was used as the dependent variable (Fig 1). The alpha level of significance was set at p = 0.05 to enter the regression model. The data were analyzed using SPSS 23.0.

## Results

The demographic, clinical, and kinematic gait parameters and the results of the EQ-5D VAS of the investigated PD cohort are presented in Table 1. In the Spearman’s analyses with the EQ-5D VAS as the dependent variable, the parameters use of assistive gait equipment (six patients used a stick, and nine patients used a walking wheel) and gait velocity showed significant correlations with the EQ-5D VAS. The results are shown in Table 2 & S1 Table. Based on a high correlation coefficient in the Spearman’s analyses, the following parameters were excluded from the block-wise regression: step duration (r = -0.43 against gait velocity, p<0.001), stride time variability (r = 0.44 against gait asymmetry, p = 0.003) and all UPDRS sub scores (r≤0.42 against the MDS-UPDRS III score, p≤0.001).

**Table 1:** Demographic, clinical, kinematic gait parameters and the Visual Analogue Scale of the Euro-QoL-5D (EQ-5D VAS) of the included patients with Parkinson’s disease (PD).

ICF Domain	Parameter	Range	Mean (if not otherwise indicated)	Standard deviation
**Outcome parameter**	EQ-5D VAS [%] (0–100)	10–85	50	8.6
**Body functions and structures**	MDS UPDRS III total (0–132)	5–67	30	12.3
	Bradykinesia subscore (0–36)	1–29	13	6
	Axial impairment subscore (0–20)	1–12	5	3
	Tremor subscore (0–28)	0–16	3	4
	Rigidity subscore (0–20)	0–16	7	4
	Gait velocity [m/s]	0.26–1.19	0.8	0.2
	Step duration [s]	0.44–0.82	0.6	0.1
	Stride time variability [%]	0.26–4.71	1.9	1.0
	Step asymmetry [%]	-20.59–36.55	7.6	6.1
**Activities**	Physical activity [MET * hours]	0.0–32.7	10	10
**Participation**	Participation in societal life (Loss of fun and interest; 0–6) °	0–6	°1	1.4
**Environmental factors**	Use of assistive gait equipment [%] °° - no equipment - walking stick - wheeled walker		65 15 20	
	Living alone [%] °°		12	
**Personal factors**	Age [years]	49–89	67	10
**Further scores used in the study**	Hoehn&Yahr (1–5) °	1–4	°2.5	0.6
	MMSE (0–30) °	17–30	°28	2
	Lachs screening questionnaire (0–15)	0–10	4	2
	BDI (0–63)	0–41	13	9

BDI, Becks Depression Inventory; ICF, International Classification for Functioning Disability and Health; MET, metabolic equivalents of task; MMSE, Mini Mental State Examination; MDS-UPDRS III, motor part of the MDS sponsored Unified Parkinson’s Disease Rating Scale.

°median

°°per cent of total.

**Table 2:** Spearman’s Rank correlation coefficients, in relation to the Visual Analogue Scale of the Euro-Qol-5D (EQ-5D VAS).

	EQ-5D VAS	
	Correlation coefficient (r)	p-value
**Age**	-0.278	0.058
**Use of assistive gait equipment**	**-0.440**	**0.002**
**Living environment**	0.064	0.674
**Participation in societal life**	-0.196	0.192
**Physical activity**	0.278	0.062
**MDS-UPDRS III**	-0.090	0.549
**Bradykinesia subscore**	-0.063	0.672
**Axial impairment subscore**	-0.227	0.125
**Tremor subscore**	0.113	0.448
**Rigidity subscore**	-0.030	0.840
**Gait velocity**	**0.295**	**0.044**
**Step duration**	0.084	0.573
**Stride time variability**	0.006	0.970
**Step asymmetry**	-0.184	0.257

Living environment: living alone vs. living with others; MDS-UPDRS III, motor part of the MDS sponsored Unified Parkinson’s Disease Rating Scale.

All remaining parameters (gait velocity, step asymmetry, physical activity, MDS-UPDRS III, loss of fun and interest in social activities, use of assistive gait equipment, living environment and age) were assigned to the ICF domains (see Statistics and Fig 1) and included in a block-wise multivariate regression analysis. The overall model explained 22.1% of the variance of the EQ-5D VAS. Use of assistive gait equipment was the only single parameter that was significantly associated with the EQ-5D VAS score. Detailed results are presented in Fig 1 and Table 3. Of note, the MDS-UPDRS III did again, after showing no relevant effect in the Spearman’s correlation analysis, not significantly contribute to the explanation of the variance of EQ-5D VAS score (Table 3).

**Table 3:** Individual values of selected parameters out of a block wise multivariate regression analysis for Health-related Quality of Life assessment in Parkinson’s disease, with consideration of International Classification of Functioning, disability and health-relevant parameters.

Model	Regression coefficient B	Standard error	Beta	T	Sig.	Part correlation	Collinearity tolerance	Collinearity VIF
**(constant)**	60.093	32.918		1.826	0.08			
**Age**	-0.395	0.279	-0.227	-1.415	0.17	-0.367	0.792	1.221
**Use of assistive gait equipment**	-10.184	4.027	**-0.413**	-2.529	**0.02**	**-0.367**	0.792	1.263
**Living environment**	16.724	8.958	0.323	1.867	0.07	0.271	0.703	1.423
**Participation in societal life**	-2.223	2.651	-0.133	-0.839	0.41	-0.122	0.838	1.193
**Physical activity (MET)**	0.252	0.284	0.140	0.886	0.38	0.129	0.845	1.183
**MDS-UPDRS III score**	0.034	0.237	0.026	0.145	0.89	0.021	0.680	1.470
**Gait velocity**	23.645	20.430	0.203	1.157	0.26	0.168	0.686	1.458
**Step asymmetry**	-0.534	0.739	-0.113	-0.723	0.48	-0.105	0.864	1.158

Significant results are printed in bold. The Visual Analogue Scale of the Euro-QoL-5D was used as parameter for HrQoL. Living environment: living alone vs. living with others; MET, metabolic equivalents of task. MDS-UPDRS III, motor part of the MDS sponsored Unified Parkinson’s Disease Rating Scale. VIF, variance inflation factor. The adjusted r^2^ of the model was 0.221.

Exclusion of participants with MMSE < 18 points did not affect these results.

## Discussion

This study embeds innovative sensor-based kinematic gait assessment into a comprehensive model in relation to HrQoL in PD. Wearable sensors are cheap, easy to handle and have the potential to substantially influence future clinical assessment and management of chronic diseases in general, and of PD in particular [9,10]. As HrQoL is an extremely important, but “difficult”, subjective concept and parameter, the conceptualization with, for example, quantitative surrogate markers seems relevant. In this respect, kinematic gait parameters are of interest, as they can be collected with the above-mentioned wearable sensors and are clearly associated with HrQoL in many chronic diseases, including PD [14–20].

As HrQoL incorporates several domains such as physical, mental, social and role functioning [6], we used the ICF framework as a comprehensive model for the statistical comparison of the kinematic gait parameters evaluated in this study with the EQ-5D VAS. The use of complex models to examine HrQoL is also supported by previous studies [16,40]. For example, a study including 210 PD patients used a path analysis based on the ICF model. They found that HrQoL in PD is strongly associated with limitations of self-care and mobility, fall history and disease duration [40]. The advantage of such complex models is that they are able to account for the interaction of a relatively large number of parameters, no matter whether they indicate a relevant association of HrQoL impairment coming from the motor [17,40,41] or the non-motor domains [14,41,42] of the disease.

Beyond gait velocity, we did not find any gait parameter significantly associated with the EQ-5D VAS in our correlation analysis. This indicates that specific, in particular kinematic, gait domains accessible with new wearable techniques do not relevantly contribute to a better understanding of HrQoL in PD. Even gait velocity may not relevantly contribute to HrQoL in PD, as it did not remain significantly associated with HrQoL in the block wise multivariate analysis. Our results are in agreement with a previous study of 236 PD patients, which also did not find a relevant association of the Parkinson’s Disease Questionnaire-39 (PDQ-39) with the time needed to walk 10 meters [17]. One explanation for these results might be the effect of the artificial environment, such as a lab or a clinic, where the tests were performed. Studies performed in a more naturalistic environment may produce different results. Indirect evidence for such an effect has been observed in datasets collected in these different environments [43]. Another explanation for these results could be that the assessment of short walks is not accurate for delineating relevant associations, and (co-occurring) symptoms such as fatigue would help delineate associations between gait and HrQoL. This assumption is indirectly supported by the results of Ellis and colleagues [17]. Here, the 6-minute walk test retained in the final model explained the chosen HrQoL parameter.

The driving parameter for HrQoL in our block-wise multivariate regression model was the use of assistive gait equipment. It is intriguing to hypothesize that this parameter is closely associated with gait velocity in PD, as the probability of reduced gait velocity has been shown to be increased by the use of an assistive gait device [44,45]. However, the r between the two parameters was only 0.35, arguing for the existence of a relevant portion of PD patients with high HrQoL choosing a low self-preferred gait velocity (or vice versa). The question remains why the use of assistive gait equipment and the EQ-5D VAS score are so closely and inversely related, as these assistive devices are prescribed to enable a more active (and safer) lifestyle. We hypothesize that the use of assistive devices is indeed associated with worse motor functionality, and our pilot study indicates that this association cannot be reflected by sensor-based parameters. As the focus of this study was not on the association between use of walking aids and HrQoL, the dataset of this study did not allow us to elaborate in more detail on this association. Future studies should focus on the investigation of the difference between the above-mentioned parameters. Moreover, they should investigate the contribution of extrinsic factors that may hinder the proper use of such aids (stairs, transport by car and public transport) and on intrinsic aspects of walking aid users. These studies may also clarify whether anxious PD patients use walking aids even when they may not necessarily physically need them. For example, it has been shown that fear of falling and uncertainty in walking lead to lowered HrQoL [46].

In line with previous results [41,47,48], neither the MDS-UPDRS III, nor its sub scores, correlated significantly with the EQ-5D VAS score. This indicates that the performance of this test does not reflect a high relevance for HrQoL in PD patients. A previous study [47] investigated 82 PD patients and found that the UPDRS III explained only 6% of the variability of the PDQ-39. Two further studies found similar results [41] [48]. Although some studies found at least some association between the UPDRS III and HrQoL (e.g., [49,50]), it is obvious that the information we can extract from clinical assessment of motor dysfunction in PD is limited. Reasons have been discussed extensively by us [7,10,51] and others [52–55], and include downsides of such assessments, such as that they are snapshots of a condition, subjective (and therefore associated with high interrater variability), and performed in an artificial environment.

There are some limitations of this study. First, the cohort investigated was relatively small. However, it covers (almost) the whole range of severity of ambulatory PD patients, and we therefore argue that the results should be generalizable to the mobile PD community. The aspect that the use of assistive gait equipment, but not gait velocity, was the most relevant factor explaining reduced HrQoL in our multivariate ICF-based model may also have relevance for the design of studies performed for other chronic diseases. Second, we did not assess anxiety or fear of falling. These parameters may be of particular relevance for our understanding of the interplay between gait difficulties and cognitive / neuropsychiatric functions. This has been shown in a previous study with elderly individuals with and without previous falls and with and without fear of falling [56]. Third, we have to emphasize that this study focused on the collection and evaluation of demographic, clinical and quantitative movement parameters; however, other HrQoL-relevant aspects, such as economic and family factors, anxiety and attitude have not been investigated. Fourth, only 15 of 43 patients used an assistive device, such as a walking stick or a walking wheel. We were thus not able to perform sub analyses concerning the influence of specific types of assistive devices due to sample size. This aspect should be considered in future studies and larger cohorts using assistive devices, as this aspect could have enormous clinical and economic implications. Finally, we cannot exclude with complete certainty increased variability of our sensor data, as we used only a single trial and did not exclude acceleration- and deceleration phases of the 20 meter walk in our analyses. The reasons for these procedures were to omit fatigue and adaptation processes, and to reflect a walking behaviour that is as close as possible to walks as performed during daily life.

In conclusion, sensor-based gait parameters do not seem to relevantly contribute to a conceptual model of HrQoL in PD. Specifically, the lack of a significant association between gait velocity and HrQoL in the ICF model is surprising and should motivate a detailed investigation of potentially independent and novel parameters. Interesting candidates are the use of assistive gait equipment, the presence of anxious behavior and complicating external factors.

## Supporting information

S1 Table Spearman’s Rank correlation matrix of all parameters. EQ-5D, Euro QoL-5D, Living environment: living alone vs. living with others; MDS-UPDRS III, motor part of the MDS sponsored Unified Parkinson’s Disease Rating Scale; VAS, visual analogue scale. P values are printed in cursive characters; significant correlations are printed in bold.
(DOCX)
