# Supplementary material for: No relevant association of kinematic gait parameters with Health-related Quality of Life in Parkinson’s disease
Source: PLoS One. 2017 May 22;12(5):e0176816. doi: 10.1371/journal.pone.0176816 (PMC5439666; doi:10.1371/journal.pone.0176816)
Supplement: S1 Table — EQ-5D, Euro QoL-5D, Living environment: living alone vs. living with others; MDS-UPDRS III, motor part of the MDS sponsored Unified Parkinson’s Disease Rating Scale; VAS, visual analogue scale. P values are printed in cursive characters; significant correlations are printed in bold. (DOCX) [file pone.0176816.s001.docx]

**Supplementary Table 1**. **Spearman’s Rank correlation matrix of all parameters**

| Correlation coefficients | EQ-5D VAS | Age | Assistive gait equipment | Living environment | Participation in societal life | Physical activity | Bradykinesia subscore | Axial impairment subscore | Tremor subscore | Rigidity subscore | MDS-UPDRS III | Gait velocity | Step duration | Stride time variability | Step asymmetry |
| --- | --- | --- | --- | --- | --- | --- | --- | --- | --- | --- | --- | --- | --- | --- | --- |
| *p-values* |  |  |  |  |  |  |  |  |  |  |  |  |  |  |  |
| EQ-5D VAS |  |  |  |  |  |  |  |  |  |  |  |  |  |  |  |
| Age | -0.278 |  |  |  |  |  |  |  |  |  |  |  |  |  |  |
|  | *0.058* |  |  |  |  |  |  |  |  |  |  |  |  |  |  |
| Assistive gait equipment | **-0.440** | 0.189 |  |  |  |  |  |  |  |  |  |  |  |  |  |
|  | ***0.002*** | *0.148* |  |  |  |  |  |  |  |  |  |  |  |  |  |
| Living environment | 0.064 | -0.007 | 0.085 |  |  |  |  |  |  |  |  |  |  |  |  |
|  | *0.674* | *0.958* | *0.532* |  |  |  |  |  |  |  |  |  |  |  |  |
| Participation in societal life | -0.196 | -0.059 | 0.158 | 0.174 |  |  |  |  |  |  |  |  |  |  |  |
|  | *0.192* | *0.682* | *0.273* | *0.231* |  |  |  |  |  |  |  |  |  |  |  |
| Physical activity | 0.278 | -0.349 | -0.278 | 0.031 | -0.150 |  |  |  |  |  |  |  |  |  |  |
|  | *0.062* | *0.014* | *0.053* | *0.838* | *0.309* |  |  |  |  |  |  |  |  |  |  |
| Bradykinesia subscore | -0.063 | -0.188 | -0.035 | -0.147 | 0.065 | -0.090 |  |  |  |  |  |  |  |  |  |
|  | *0.672* | *0.154* | *0.792* | *0.279* | *0.654* | *0.544* |  |  |  |  |  |  |  |  |  |
| Axial impairment | -0.227 | 0.041 | 0.292 | -0.094 | 0.103 | -0.135 | **0.417** |  |  |  |  |  |  |  |  |
|  | *0.125* | *0.757* | *0.025* | *0.492* | *0.478* | *0.360* | ***0.001*** |  |  |  |  |  |  |  |  |
| Tremor subscore | 0.113 | -0.186 | -0.048 | -0.247 | 0.099 | 0.146 | 0.166 | -0.140 |  |  |  |  |  |  |  |
|  | *0.448* | *0.159* | *0.717* | *0.066* | *0.492* | *0.323* | *0.209* | *0.291* |  |  |  |  |  |  |  |
| Rigidity subscore | -0.030 | 0.002 | 0.060 | -0.158 | 0.202 | -0.003 | **0.339** | 0.126 | **0.403** |  |  |  |  |  |  |
|  | *0.840* | *0.988* | *0.650* | *0.244* | *0.160* | *0.981* | ***0.009*** | *0.341* | ***0.002*** |  |  |  |  |  |  |
| MDS-UPDRS III | -0.090 | -0.100 | 0.111 | -0.217 | 0.197 | -0.075 | **0.829** | **0.498** | **0.424** | **0.636** |  |  |  |  |  |
|  | *0.549* | *0.450* | *0.402* | *0.109* | *0.170* | *0.615* | ***0.000*** | ***0.000*** | ***0.001*** | ***0.000*** |  |  |  |  |  |
| Gait velocity | 0.295 | -0.182 | **-0.350** | -0.158 | -0.014 | 0.346 | -0.096 | -0.187 | 0.109 | -0.095 | -0.076 |  |  |  |  |
|  | *0.044* | *0.165* | ***0.006*** | *0.245* | *0.923* | *0.015* | *0.471* | *0.157* | *0.413* | *0.474* | *0.567* |  |  |  |  |
| Step duration | 0.084 | 0.062 | 0.186 | 0.175 | 0.151 | -0.066 | 0.194 | 0.225 | 0.088 | 0.027 | 0.220 | **-0.430** |  |  |  |
|  | *0.573* | *0.637* | *0.156* | *0.196* | *0.294* | *0.652* | *0.140* | *0.087* | *0.507* | *0.838* | *0.093* | ***0.001*** |  |  |  |
| Stride time variability | 0.006 | -0.067 | -0.060 | -0.132 | -0.150 | 0.293 | 0.078 | 0.038 | 0.175 | -0.033 | -0.042 | -0.191 | 0.091 |  |  |
|  | *0..970* | *0.639* | *0.675* | *0.372* | *0.337* | *0.059* | *0.592* | *0.792* | *0.225* | *0.820* | *0.771* | *0.179* | *0.525* |  |  |
| Step asymmetry | -0.184 | -0.155 | 0.172 | 0.223 | 0.021 | -0.039 | 0.185 | 0.109 | -0.017 | -0.042 | 0.130 | **-0.278** | 0.170 | **0.435** |  |
|  | *0.275* | *0.278* | *0.226* | *0.128* | *0.895* | *0.805* | *0.199* | *0.450* | *0.622* | *0.772* | *0.369* | ***0.048*** | *0.232* | ***0.003*** |  |

EQ-5D, Euro QoL-5D, Living environment: living alone vs. living with others; MDS-UPDRS III, motor part of the MDS sponsored Unified Parkinson’s Disease Rating Scale; VAS, visual analogue scale. P values are printed in cursive characters; significant correlations are printed in bold.
